# Cowden Syndrome With Gall Bladder Polyps and Incidental Gall Bladder Carcinoma

**DOI:** 10.7759/cureus.39794

**Published:** 2023-05-31

**Authors:** Praveen Agarwal, Ashish Sachan, Vivek Goel, Sourabh Jindal, Pradeep Jain

**Affiliations:** 1 Surgical Gastroenterology, Fortis Hospital, Shalimar Bagh, Delhi, IND

**Keywords:** radical cholecystectomy, ileal pouch–anal anastomosis, intestinal polyps, incidental gall bladder adenocarcinoma, gall bladder polyps, pten gene, cowden syndrome

## Abstract

Cowden syndrome is an uncommon autosomal dominant disorder characterized by multiple hamartomas in various tissues. It is associated with germline mutation in the phosphatase and tensin homolog (PTEN) gene. It has an increased risk of malignancies of various organs (commonly breast, thyroid, and endometrium) and benign overgrowth of tissues like skin, colon, and thyroid. Here, we present a case of Cowden syndrome in a middle-aged female who presented with acute cholecystitis with gall bladder polyps along with intestinal polyps. She underwent total proctocolectomy with ileal pouch-anal anastomosis (IPAA) with diversion ileostomy and cholecystectomy, which was further proceeded to completion of radical cholecystectomy based on the final histopathology report as incidental gall bladder carcinoma. To the best of our knowledge, this association is seen for the first time in the literature. In Cowden syndrome, patients should be counseled for regular follow-up and instructed to be aware of the signs and symptoms of different types of cancers with higher incidence.

## Introduction

Cowden syndrome (CS) is an uncommon autosomal dominant disorder characterized by multiple hamartomas in various tissues [1,2]. The incidence is one in 200000 people [3]. It is associated with germline mutation in the phosphatase and tensin homolog (PTEN) gene. PTEN is a tumor suppressor gene located on chromosome 10q23.3 [1,2]. CS is an uncommon autosomal dominant disorder characterized by multiple hamartomas in various tissues.

Eighty percent (80%) of patients with CS have been identified with this germline mutation [3]. It is associated with multiple hamartomatous neoplasms of the skin, oral mucosa, gastrointestinal tract, bones, central nervous system, eyes, and genitourinary tract [4]. It has increased risk of malignancies of various organs (particularly the breast, thyroid, and endometrium) and benign overgrowth of tissues like the skin, colon, and thyroid [2,5]. Due to the risk of multiple malignancies, patients may present with a variety of clinical signs and symptoms. Mucocutaneous lesions, which are considered pathognomonic for CS, often go unnoticed [6].

Here, we want to discuss a case of CS in a middle-aged female patient who presented with features of acute cholecystitis with multiple gall bladder polyps but whose histology report later revealed gall bladder cancer. To the best of our knowledge, this association has been seen for the first time in literature.

## Case presentation

**Figure 1:**
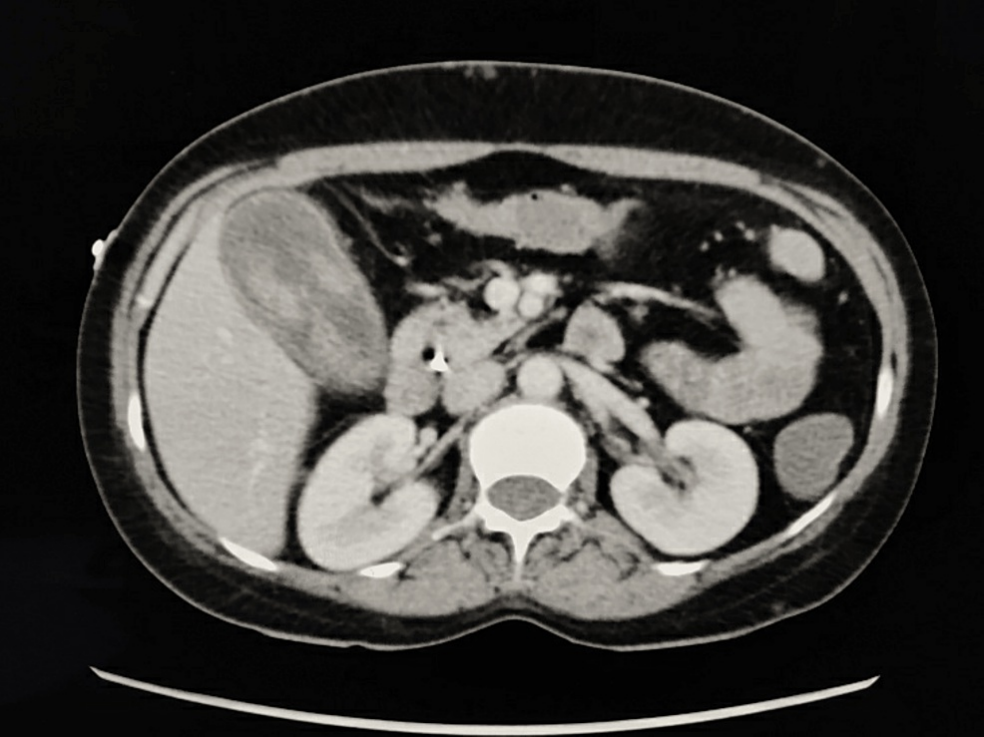
CECT abdomen image showing the gall bladder with multiple iso- to mildly hyperdense non-enhancing lesions – likely gall bladder sludge/calculi CECT: contrast-enhanced computed tomography

A 38-year-old diabetic, normotensive female presented with complaints of pain in the right upper abdomen and vomiting for one week. There was no history of fever with no signs of cholestasis. Her ultrasonography (USG) of the whole abdomen suggested acute cholecystitis with multiple gall bladder polyps and mild pancreatitis. Computed tomography (CT) of the whole abdomen showed multiple atypical hemangiomas of the liver, a dilated common bile duct (CBD) (10 mm), and a well-distended gall bladder with multiple iso- to mildly hyperdense non-enhancing lesions-likely gall bladder sludge or calculi (Figure 1).

**Figure 2:**
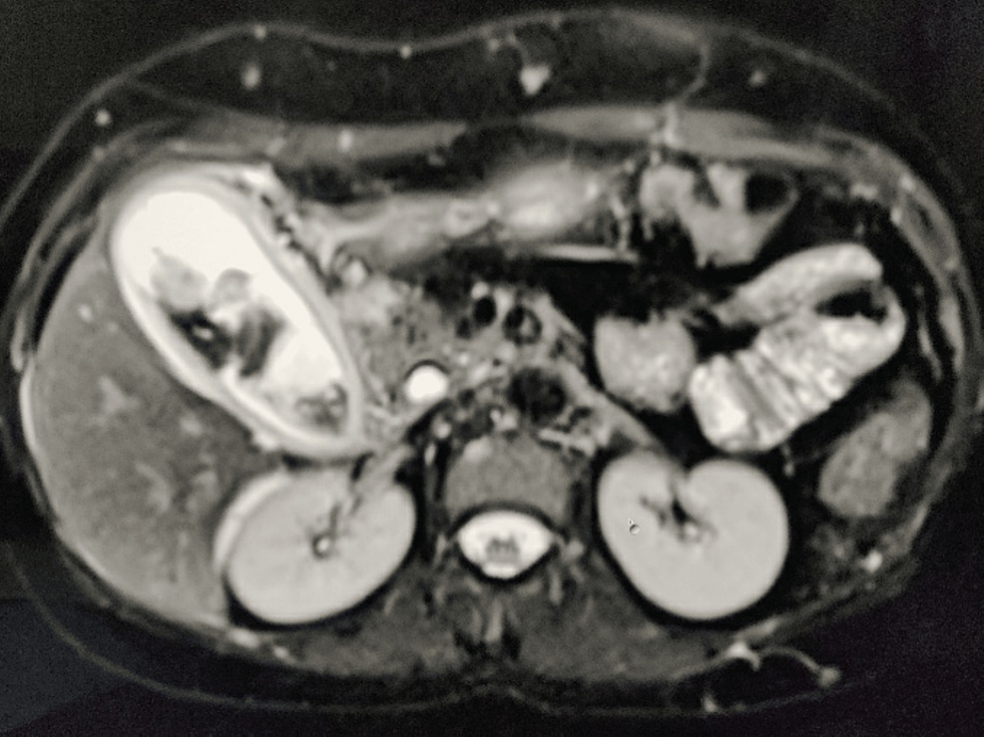
MRCP image showing the gall bladder with heterogenous contents likely sludge with thickened gall bladder wall with pericholecystic fluid collection MRCP: magnetic resonance cholangiopancreatography

Magnetic resonance cholangiopancreatography also showed multiple hemangiomas in both lobes of the liver, features of acute cholecystitis with gall bladder sludge, dilated CBD with sludge, and pancreatitis (Figure 2).

**Figure 3:**
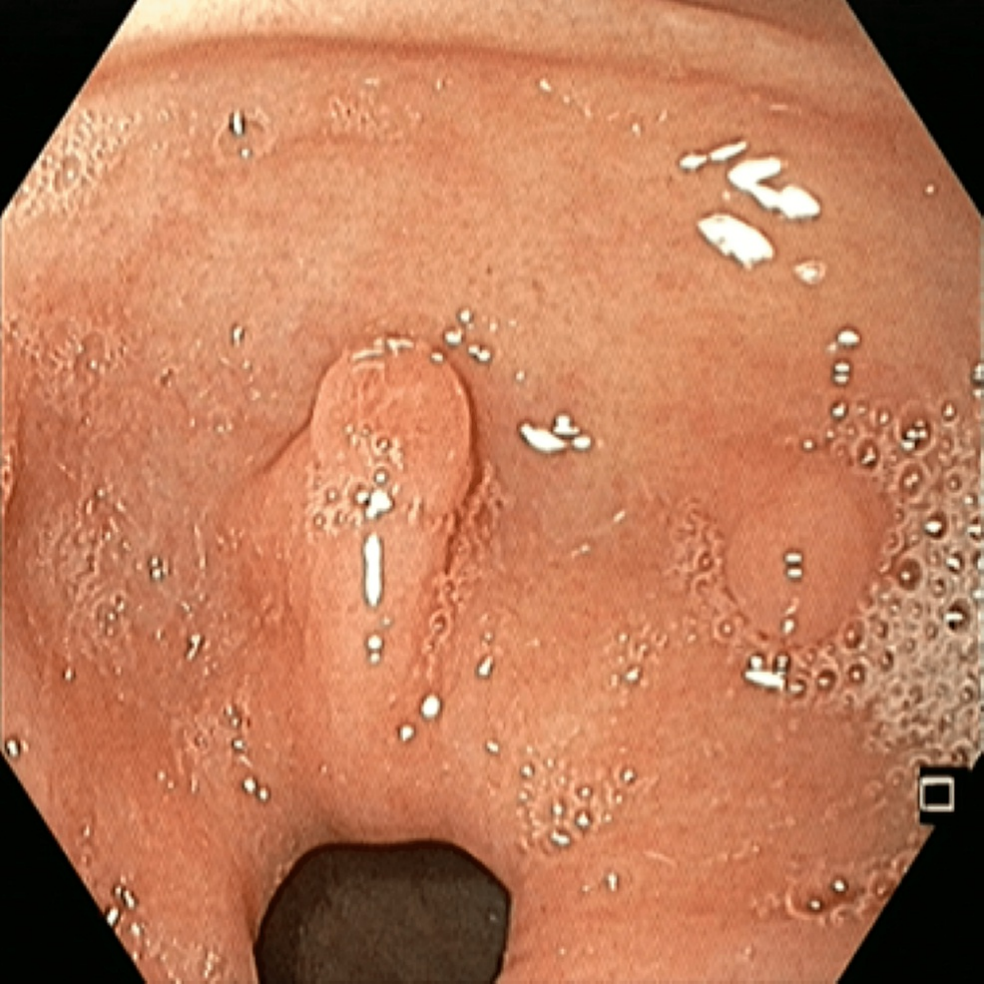
UGIE showing multiple variable-sized polyps in the stomach UGIE: upper gastrointestinal endoscopy

Her CA 19.9 and serum CEA were 42.59 U/ml and 0.05 ng/ml, respectively. Endoscopic retrograde cholangiopancreatography (ERCP) showed sludge in the common bile duct (CBD); after CBD clearance, a 7Fr plastic stent was placed. While passing the side view endoscope, multiple variable-sized polyps were found in the stomach and second part of the duodenum (Figure 3).

**Figure 4:**
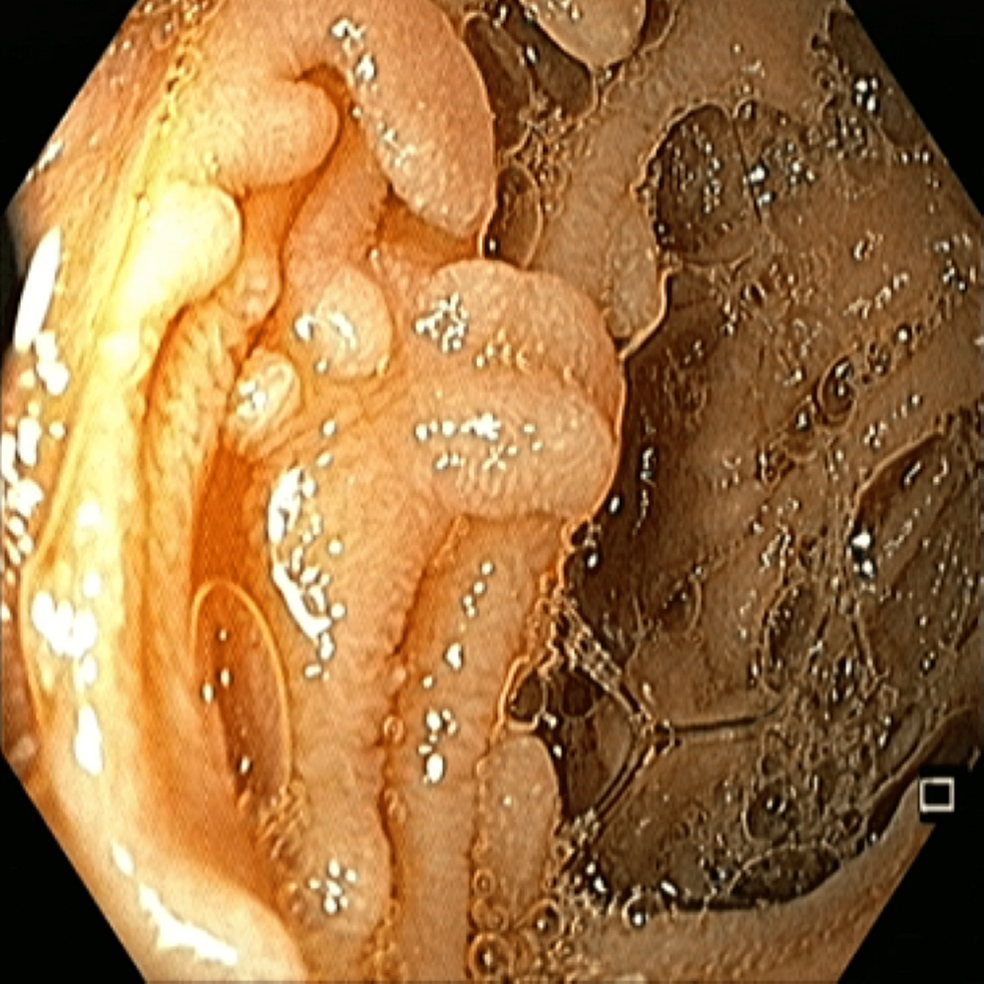
Colonoscopy showing multiple sessile and pedunculated polyps in the entire colon and rectum (largest 1.5 cm x 1 cm)

A polypectomy was done and its histopathology was a low-grade dysplastic adenomatous polyp. CT enterography revealed multiple polyps in the fundus and body region of the stomach, jejunum, and terminal ileum (the largest one measuring 20 x 13 mm). Colonoscopy showed multiple sessile and pedunculated polyps (<100) in the entire colon and rectum (largest 1.5 cm x 1 cm) (Figure 4).

**Figure 5:**
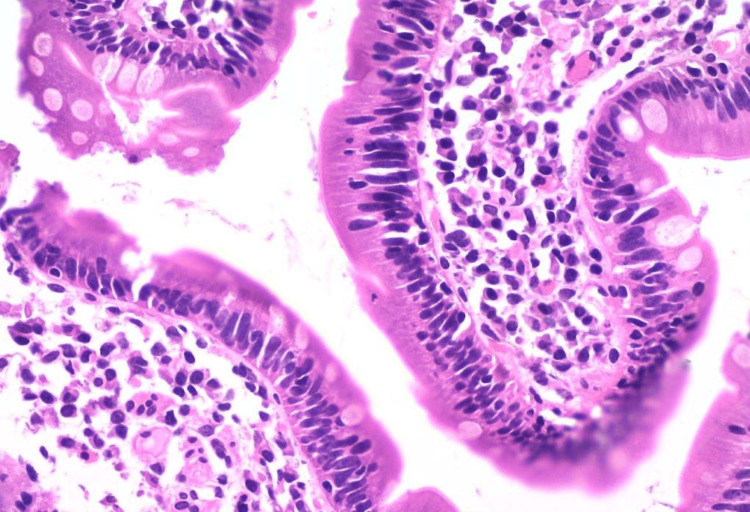
HPE image of the excised polyp showing a mild dysplastic adenomatous polyp HPE: histopathological examination

Histopathological examination (HPE) of the excised polyp revealed a mild dysplastic adenomatous polyp (Figure 5).

Whole-body positron emission tomography (CT) was done to see the involvement of other organs. It revealed multiple, ill-defined intraluminal soft tissue lesions in the gall bladder, with one of the lesions showing significant hypermetabolism-likely inflammatory. It also showed hypermetabolic, heterogeneously enhancing multiple, variable-sized soft tissue density nodular lesions involving both the breast parenchyma and multinodular goiter. USG-guided fine-needle aspiration (FNA) cytology of both breast lesions was done, which showed bilateral fibroadenoma. Based on all these findings, genetic mutational analysis with next-generation sequencing was done, which confirmed the heterozygous variant in exon 5 of the PTEN gene on chromosome 10. An “A” to “G” substitution was detected at nucleotide position 275, leading to a change in the amino acid sequence from aspartic acid to glycine at codon 92. These variants were detected as related to the clinical phenotype (CS and Lhermitte-Duclos syndrome). Hence, a diagnosis of CS was made. Retrospectively, we also noticed skin and tongue lesions suggestive of CS.

A focused history and medical examination of other members of the patient’s family were done, but there were no significant clinical findings. The family members were counseled to have a genetic analysis, but they refused.

**Figure 6:**
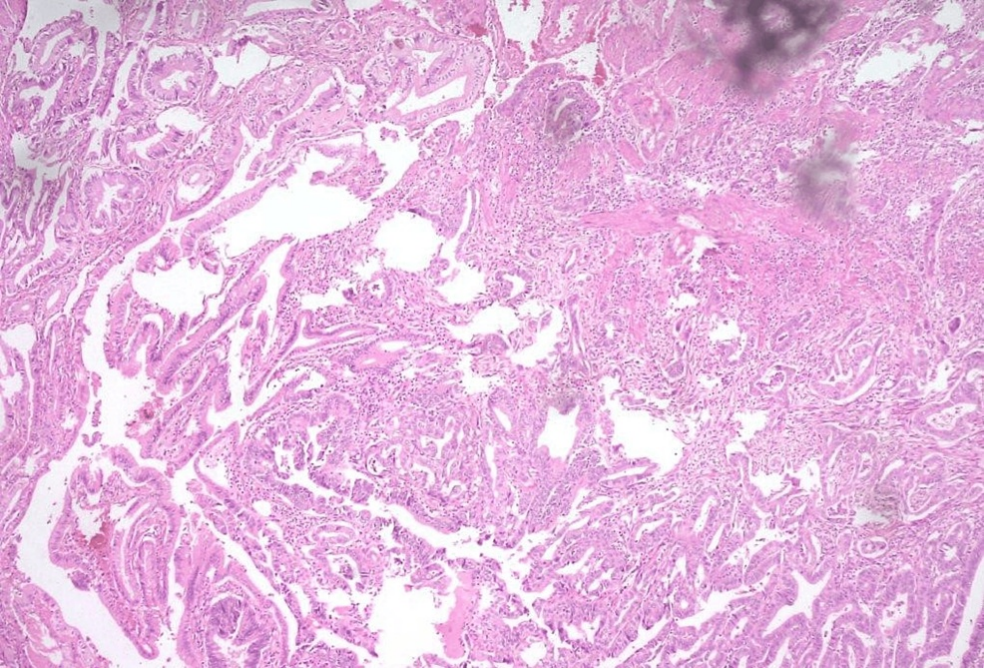
HPE image of the gall bladder specimen showing invasive, well-differentiated adenocarcinoma of the gall bladder HPE: histopathological examination

Because of dysplastic polyps in the colon and rectum, she underwent laparoscopic total proctocolectomy with ileal pouch-anal anastomosis with diversion ileostomy and cholecystectomy. Additionally, the patient was also counseled to undergo a prophylactic hysterectomy, but she refused. The HPE report of the proctocolectomy specimen showed adenomatous polyps with mild to moderate dysplasia; there was no invasive carcinoma in any of the polyps examined. A well-differentiated adenocarcinoma (pT1bNxMx) with a negative cystic duct margin in the gall bladder was a surprising result on histopathology (Figure 6).

The case was discussed in the tumor board, and she underwent laparoscopic completion radical cholecystectomy after 10 days of primary surgery. The histopathology report showed no involvement of the underlying liver or cystic duct stump, with no nodal metastasis. The postoperative period was uneventful. After three months, ileostomy closure was done. During follow-up, the patient was advised to undergo prophylactic subcutaneous mastectomy and thyroidectomy, but she refused. Now the patient is on regular follow-ups with USG for breast and thyroid, upper gastrointestinal endoscopy, pouchoscopy, and CECT abdomen.

## Discussion

CS is a rare hereditary cancer syndrome, named after Cowden’s family, and documented with signs of disease in 1963 by Lloyd and Dennis [7,8]. It is part of the PTEN hamartoma tumor syndrome, which also consists of Bannayan-Riley-Ruvalcaba syndrome, Lhermitte-Duclos disease, Proteus syndrome, and Proteus-like syndrome. Only CS is associated with a documented predisposition to malignancies, with a slight female predominance [8,9]. The age of the onset varies from four to 75 years [10].

The loss of PTEN function can lead to cancer development in various organs. In almost 100% of cases, mucocutaneous lesions (trichilemmomas, papillomatous papules, and acral keratosis) are seen [9,11]. These lesions are pathognomic and the first signal in CS patients for early diagnosis before malignancy develops. Breast lesions are found in 76% of all cases as fibrocystic disease [8], with breast carcinoma occurring in 25-50% of female cases. The most frequent extracutaneous manifestation of CS is thyroid disease, present in 75% of cases [8]. The most common malignancy seen in CS is breast cancer, followed by thyroid cancer (3-10% cases) [11,12].

Gastrointestinal polyps are mainly found in the rectum and sigmoid in 40% of patients, but there is no documented evidence of gall bladder polyps or gall bladder cancer in the literature [9,11]. These polyps are predominantly hamartomatous colorectal polyps. Until recently, it was believed that gastrointestinal involvement was not cancerous. But studies have reported that patients with CS are at increased risk for cancer [13]. Endometrial cancer also occurs in 5-10% of females with the disease [11].

The criteria for the diagnosis of CS have been updated several times. In 2013, an evidence-based review led to the revision of diagnostic criteria, which were adopted by the U.S. National Comprehensive Cancer Network (NCCN) [14].

The management of patients with CS involves the early detection of malignant conditions, patient education, genetic counseling, and regular surveillance [15]. The NCCN provides updated guidelines for men and women with CS that outline a cancer surveillance program [12].

CS, as mentioned above, may involve malignancies in various organs; therefore, aggressive diagnostic evaluation and follow-up should be done in suspected cases. This helps in the timely diagnosis and early treatment of the disease.

Lastly, our patient demonstrated a much wider spectrum of imaging findings, including multinodular goiter of the thyroid, fibroadenoma of both breasts, multiple liver hemangiomas, gastrointestinal adenomatous polyps, and a new finding of gall bladder polyps and gallbladder carcinoma. After an extensive literature search, we identified that this is the first case report of a gall bladder polyp and carcinoma gall bladder in a patient with CS.

## Conclusions

CS patients usually present with intestinal polyps, but in our case, the initial evaluation suggested gall bladder polyps along with intestinal polyps, which turn out to be adenocarcinoma of the gall bladder on histopathological examination. Hence, even with benign lesions on evaluation, the potential for malignant lesions must be kept in mind, and a frozen section biopsy should be considered during surgery.

In CS, the patient should be counseled for regular follow-up and instructed to be aware of the signs and symptoms of different types of cancers with a higher incidence.
